# Neural Engineering for Rehabilitation

**DOI:** 10.1155/2017/9638098

**Published:** 2017-04-30

**Authors:** Han-Jeong Hwang, Do-Won Kim, Janne M. Hahne, Jongsang Son

**Affiliations:** ^1^Department of Medical IT Convergence Engineering, Kumoh National Institute of Technology, Gumi 730-701, Republic of Korea; ^2^Department of Biomedical Engineering, Chonnam National University, Yeosu 59626, Republic of Korea; ^3^Department of Neurorehabilitation Engineering, University Medical Center Göttingen, Georg-August University Göttingen, 37075 Göttingen, Germany; ^4^Sensory Motor Performance Program, Rehabilitation Institute of Chicago, Chicago, IL 60611, USA; ^5^Department of Physical Medicine & Rehabilitation, Northwestern University, Chicago, IL 60611, USA

Neural engineering (also called neuroengineering) is an interdisciplinary research area, and its fundamental goal is to understand underlying mechanisms of the nervous system and to provide rehabilitative solutions for the treatment of neurological disorders, such as autism, stroke, multiple sclerosis, epilepsy, and Alzheimer's and Parkinson's disease. So far, neural engineering has played a crucial role in advancing neurorehabilitation technologies by developing novel assistive and rehabilitation tools/methods/systems, thereby providing more effective and efficient rehabilitation. This special issue aims at sharing the current state-of-the-art trends and future directions in the field of neural engineering for rehabilitation, and it includes eight up-to-date articles related to neurorehabilitation. The selected articles cover a wide range of neurorehabilitation research topics: neurofeedback, brain-computer interface (BCI), hybrid BCI, transcranial direct current stimulation (tDCS), thermotherapy, robot-aided neurorehabilitation, relation between the features of olfactory stimuli and electroencephalography (EEG), and diagnosis of autism spectrum disorder based on EEG.

In the article entitled “Data-Driven User Feedback: An Improved Neurofeedback Strategy considering the Interindividual Variability of EEG Features” by C.-H. Han et al., the authors proposed data-driven user feedback to improve a neurofeedback strategy considering the interindividual variability of EEG features. In the experiment, subjects performed a meditation paradigm, where a babbling brook sound, a picture of a beautiful valley, and a quiet pure-tone beep sound with a period of three seconds were simultaneously provided to each subject. By dividing the entire range of an EEG feature into a lot of bins using nonuniform bin sizes, the authors could optimize bin sizes and permit users to experience a wider range of feedback without a customization process. Each individual experienced significantly increased feedback levels, which were 139% and 144% of the original levels with uniform bin sizes in the offline and online experiments, respectively. It is expected that the proposed method could effectively increase the overall range of feedback levels, thereby providing an improved neurofeedback strategy.

The article entitled “Vowel Imagery Decoding Toward Silent Speech BCI Using Extreme Learning Machine with Electroencephalogram” by B. Min et al. investigated four different classifiers to decode imagined speech based on EEG signals, where five vowels, /a/, /e/, /i/, /o/, and /u/, were tested. The authors employed four EEG features (mean, variance, standard deviation, and skewness) and reduced the dimensionality of the feature vector using a sparse regression model. For classification, they tested two variants of support vector machine (SVM) and of an extreme learning machine. As a result, the extreme learning machine showed a mean accuracy of about 70% for all pairwise classification, which was better than the SVM's variants. The results could be utilized to enhance the performance of imagined-speech-based BCIs and fundamentally contribute to increasing the quality of life for patients with neurological disorders.

The article entitled “Evaluation of a Compact Hybrid Brain-Computer Interface System” by J. Shin and colleagues suggested a way of realizing a compact hybrid EEG and near-infrared spectroscopy (NIRS) acquisition system using a portable NIRS device with an economic EEG system. To prove its feasibility, the authors conducted a typical BCI experiment in which subjects performed a mental arithmetic (MA) task. They classified MA tasks from baseline and compared the classification accuracies obtained using each of the modalities (EEG or NIRS) and the hybrid system. The hybrid EEG and NIRS system showed an increase of performance compared to the unimodal EEG and NIRS systems by 6.2% and 2.5%, respectively, demonstrating the feasibility of the implemented hybrid system. As the proposed hybrid system is based on portable platforms, its potential use is not confined to a laboratory environment and can be extended to real-life situations, such as neurorehabilitation.

The article entitled “Effect of Anodal-tDCS on Event-Related Potentials: A Controlled Study” by A. Izzidien et al. investigated the impact of anodal tDCS on three different EEG phenomena that are event-related potential (ERP), synchronization (ERS), and desynchronization (ERD). The authors showed that absolute ERP power significantly increased after tDCS stimulation, but not for ERD and ERS, concluding that tDCS may help enhance the accuracy of ERP-based BCI spellers for patients with neurological disorders. Since the ERP power was significantly improved at Pz, tDCS stimulation may help the development of neurorehabilitation methods, especially targeting the parietal lobe.

In the article entitled “Integrative Evaluation of Automated Massage Combined with Thermotherapy: Physical, Physiological, and Psychological Viewpoints” by D.-W. Kim et al., the effect of massage therapy was investigated alone and in combination with infrared heating on physical, physiological, and psychological aspects. Various physical, physiological, and psychological changes were observed, mostly showing significantly positive effect on physical functioning, increased parasympathetic response, and decreased psychological stress and anxiety, especially when massage therapy was combined with infrared heating. The results indicate that thermotherapy could lead to enhanced physical functions during rehabilitation.

The article entitled “Patient-Centered Robot-Aided Passive Neurorehabilitation Exercise Based on Safety-Motion Decision-Making Mechanism” by L. Pan et al. presented a novel motion control approach for patient-centered robot-aided passive neurorehabilitation exercise from the safety point of view. The proposed approach included observing and assessing the physical state of training impaired-limb and motion performances and regulating training parameters (e.g., motion speed and training range) in real time. The authors demonstrated the efficacy of the suggested control strategy in two experiments performed with healthy subjects and stroke patients. The research results could be used to design a rehabilitation program, ensuring the safety of patients for emergency events.

The article entitled “Analysis of the Influence of Complexity and Entropy of Odorant on Fractal Dynamics and Entropy of EEG Signal” by H. Namazi et al. analyzed the relationship between olfactory characteristics and EEG features. The authors used five pleasant odorants and investigated how their molecular complexity and entropy were related to EEG features. The authors found that the complexity of the EEG is positively coupled with the molecular complexity of the odorant, but the entropy of EEG showed an opposite trend. Understanding the relation between external stimuli (olfactory in this study) and brain reaction could help the development of rehabilitation therapy for different brain diseases.

The article entitled “EEG-Based Computer-Aided Diagnosis of Autism Spectrum Disorder Using Wavelet, Entropy, and ANN” by R. Djemal et al. proposed a diagnosis method of autism spectrum disorder based on machine-learning approach. The authors used discrete wavelet transform (DWT) and entropy as features and artificial neural network as a classifier. The highest diagnosis accuracy was obtained (96%) when using standard deviations of extracted DWT coefficients. The results could be used to diagnose autism patients more accurately, providing an appropriate rehabilitation method for autism spectrum disorder.

In this special issue, we provide the eight research articles showing recent advances in neural engineering for rehabilitation. We hope that this special issue will further contribute to promoting the development of neurorehabilitation and fundamentally providing clinically feasible neurorehabilitative methods. To this end, more clinical studies with real patients are especially required to accurately evaluate the clinical effect of new rehabilitative methods even though experiments performed with healthy subjects generally show similar clinical effects.

*Han-Jeong Hwang* *Han-Jeong Hwang* *Do-Won Kim* *Do-Won Kim* *Janne M. Hahne* *Janne M. Hahne* *Jongsang Son* *Jongsang Son*

